# Critically ill patients with infectious diseases – clinical, evolutive and etiological issues

**DOI:** 10.1186/1471-2334-14-S7-P22

**Published:** 2014-10-15

**Authors:** Irina Niculescu, Augustin Cupşa, Florentina Dumitrescu, Iulian Diaconescu, Livia Dragonu, Dan Hurezeanu, Mariana Stănescu, Mihai Jianu

**Affiliations:** 1 “Victor Babeş” Clinical Hospital of Infectious Diseases and Pneumology, Craiova, Romania; 2 University of Medicine and Pharmacy Craiova, Romania

## Background

Infectious diseases (ID) are the second cause of death after cardiovascular diseases worldwide; severe sepsis and septic shock, syndromes induced by infection have an increasing incidence and high mortality requiring diagnosis and intensive therapy and appropriate attitude.

Objectives: to establish the clinical, evolutive and etiological aspects of ID evolving critically ill patients with ID.

## Methods

Retrospective study (January 2013-March 2013) on 96 critically ill patients with ID hospitalized in the Intensive Care Unit of the "Victor Babeş" Hospital Craiova.

## Results

General data of the study lot: gender distribution was balanced (M:F = 1:1), 75% of patients were from urban areas and the median age was 60 (IQR: 1-95) years. ID were: respiratory infection (67.71%), gastrointestinal (12.50%), urinary (9.37%), neurological (9.37%) and cardiovascular (1.05%). Failure or organ dysfunction (one or combination) in the study group: respiratory (69.79%), neurological (9.37%), renal (5.21%), liver (1.04%), hematologic (1.04%) and heart (1.04%). Sepsis was diagnosed in 35.42% of patients, severe sepsis in 22.92% and 5.21% developed septic shock.

Comorbidities were identified in 88.54% of patients (one or combination) as follows: chronic cardiovascular diseases – 39.58% of patients, neuropsychiatric – 16.67%, pulmonary – 12.5%, obesity – 10.42%, HIV – 9.37%, urogenital – 8.33%, diabetes and cancer – 7.29% each, other – 11.46%. Favorable outcome was recorded in 88.54% of cases, death was recorded in 11.46% of cases. The etiology had been identified in 32.29% of the cases, as follows: bacterial etiology – 74.20% of the cases, viral and parasitic each in 12.90%. The etiology of ID was represented by: Gram-negative bacteria (38.71%), Gram-positive bacteria (22.58%), *Mycobacterium tuberculosis* and *Toxoplasma gondii* (12.90% in each case), measles virus (9.68%) and pdm2009 influenza virus (3.23%).

## Conclusion

Critically ill patients with ID presented most commonly respiratory or gastrointestinal infection and most frequently developed respiratory or neurological failure or dysfunction. Most critically ill patients with ID associated comorbidities, most commonly chronic cardiovascular diseases. Evolving critically ill patients with ID had significantly increased risk of death. Gram-negative bacteria dominated the etiology of ID in critically ill patients, followed by Gram-positive bacteria.

